# Decreased expression of alpha-2-HS glycoprotein in the sera of rats treated with *Eurycoma longifolia* extract

**DOI:** 10.3389/fphar.2015.00211

**Published:** 2015-09-23

**Authors:** Yeng Chen, Wai-Mei Phang, Alan K.-W. Mu, Choon-Keat Chan, Bin-Seng Low, Sreenivasan Sasidharan, Kit-Lam Chan

**Affiliations:** ^1^Department of Oral Biology and Biomedical Sciences, Faculty of Dentistry, University of MalayaKuala Lumpur, Malaysia; ^2^Oral Cancer Research and Coordinating Centre, Faculty of Dentistry, University of MalayaKuala Lumpur, Malaysia; ^3^Department of Cell and Molecular Biology, Faculty of Biotechnology and Biomolecular Sciences, Universiti Putra MalaysiaSelangor, Malaysia; ^4^Malaysian Institute of Pharmaceuticals and Nutraceuticals, Ministry of Science, Technology and InnovationPulau Pinang, Malaysia; ^5^School of Pharmaceutical Sciences, Universiti Sains MalaysiaPulau Pinang, Malaysia; ^6^School of Medicine, Taylor’s UniversitySelangor, Malaysia; ^7^Institute for Research in Molecular Medicine, Universiti Sains MalaysiaPulau Pinang, Malaysia

**Keywords:** 2-dimensional electrophoresis, alpha-2-HS glycoprotein, *Eurycoma longifolia*, plant extract, mouse model

## Abstract

*Eurycoma longifolia* is a Malaysian native herb that has been widely used as an aphrodisiac and a remedy for andropause. Although the physiological effects of the plant extract were predicted as a result of the alterations in protein expression, the key protein(s) involved in these alterations are still unclear. In the present study, we have investigated the effect of standardized *E. longifolia* extract on serum protein expression up to 28 days following oral administration in rats. Serum protein profiles were analyzed by 2-dimensional electrophoresis, and altered proteins were identified via mass spectrometry. We observed that alpha-2-HS glycoprotein (AHS) was significantly decreased in the serum of experimentally treated rats compared to pre-treated animals. Moreover, reduction in AHS was confirmed using competitive enzyme-linked immunosorbent assay. AHS expression is known to be associated with insulin resistance and diabetes. Our data indicated that serum AHS was reduced in rats treated with standardized *E. longifolia* extract, and therefore form a prelude for further investigation into the effects of this natural extract in animal models involving infertility and diabetes.

## Introduction

*Eurycoma longifolia* Jack is a tall, slender shrub-tree that is commonly found in the lowland forests up to 500 m above sea level in Southeast Asia (Kuo et al., 2004). The root of *E*. *longifolia* is widely used in folk medicine as a recuperative tonic after childbirth and for treating fever, boils, wound ulcers, syphilis, and bleeding gums (Burkill, 1966). It has also been employed as a traditional remedy for malaria, high blood pressure, fatigue, loss of sexual desire, and impotence (Chan et al., 1998). Notably, it was demonstrated that eurycomanone, a quassinoid isolated from *E. longifolia* roots, displayed cytotoxicity against cancer cells as well as antimalarial and antiulcer activities (Bhat and Karim, 2010). Furthermore, several studies showed that *E. longifolia* extracts could improve sexual performance (Ang and Sim, 1998; Zanoli et al., 2009) and sperm quality in rats (Noor et al., 2004; Chan et al., 2009; Wahab et al., 2010). Clinical studies confirmed that sperm quality and testosterone levels in infertile males improved upon treatment with *E. longifolia* (Tambi and Imran, 2010; Tambi et al., 2012).

**FIGURE 1:**
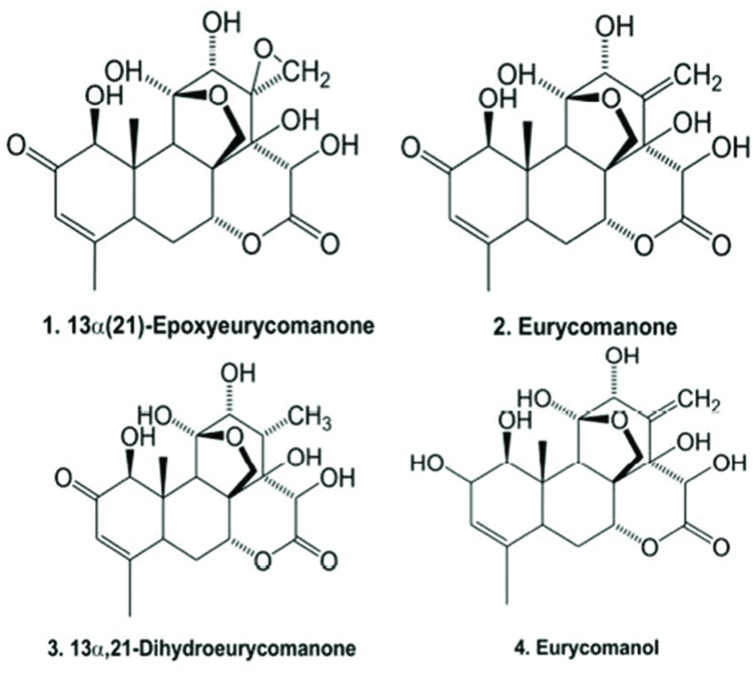
**The active ingredients of *Eurycoma longifolia* F2 extract**.

We recently provided evidence that eurycomanone obtained from the standardized extract F2 could improved spermatogenesis and fertility in rats (Low et al., 2013). The validated HPLC method for the phytochemical analysis of the active constituents in the standardized extract F2 was found to contain 34.14% w/w of the major bioactive quassinoids, comprising 13α(21)-epoxyeurycomanone (1) (7.39 ± 0.17%, w/w), eurycomanone (2) (14.49 ± 0.26%, w/w), 13,21-dihydroeurycomanone (3) (0.72 ± 0.06%, w/w) and eurycomanol (4) (9.54 ± 0.22%, w/w) (**Figure 1**) (Low et al., 2013). In fact, we observed elevation of testosterone, luteinizing hormone, and follicle stimulating hormone in plasma, along with suppression of estrogen, after oral administration of *E. longifolia* extract, revealing its effect on the hypothalamic-pituitary-gonadal axis (Low et al., 2013). This increase in male fertility and hormones following treatment with the quassinoid-rich *E. longifolia* extract suggests that it may alter animal physiology. However, no study has identified the protein(s) linking eurycomanone to its *in vivo* physiological effects. Although it is predicted that these physiological changes result from alterations in protein expression following *E. longifolia* treatment, it remains unclear which proteins might be affected (Tsai and Wiltbank, 1998). Thus, the aim of this study was to investigate serum proteomic profile changes following administration of standardized bioactive *E. longifolia* in an animal model. *E. longifolia*-treated male Sprague-Dawley rats were compared to pre-treated animals over 28 days, and serum proteins were analyzed using two-dimensional gel electrophoresis (2-DE) and subsequently identified via mass spectrometry (MS).

## Materials and Methods

### Extraction

The standardized *E. longifolia* extract was prepared and quassinoid content was determined through a high performance liquid chromatography (HPLC)-based method as described previously (Teh et al., 2011; Chan et al., 2012). *E. longifolia* roots were purchased from Biotropics Malaysia Berhad, Kuala Lumpur, Malaysia. A voucher specimen of the plant has been deposited at Penang Botanical Garden, Malaysia, with a reference no. of JTB1189 (Low et al., 2013). Briefly, the *E*. *longifolia* roots were ground into a powder, and ∼100 kg were extracted with 10 L of methanol at 45°C (8 h per day, replacing fresh solvent for five consecutive days). Filtrates of the crude extract were combined and evaporated to dryness *in vacuo*. The dried methanolic extract was subjected to a resin-packed column and gradually eluted with a MeOH–H_2_O mixture (1:0–0:1 ratio; Chan et al., 2012). The quassinoid-containing fraction was identified as the standardized *E. longifolia* extract and was dried before oral administration.

### Animals

Six Sprague-Dawley male rats (12 weeks old, weighing 290–310 g) were obtained from the Animal Research and Service Centre, University of Science Malaysia, Penang, Malaysia. The animals were fed with a commercial pellet diet (Gold Coin, Penang, Malaysia) and the water was provided *ad libitum*. The rats were individually caged and housed in a 12 h light-dark cycle at ambient room temperature. All animal experimentation was humanely conducted and maintained in accordance with Guide for the Care and Use of Laboratory Animals 2010 Animal Research and Service Centre, Universiti Sains Malaysia and European legislation (EEC n. 86/609). The experimental designs and procedures were approved by the institutional Animal Ethics Committee with Approval No: USM/Animal Ethics Approval/2012/(79) (386).

### Treatment and Monitoring

Prior to experimentation, the rats were kept for 1 week upon husbandry in accordance with guidelines set by the European Agency for the Evaluation of Medicinal Products. Animals were kept in cages with free access to food and water and were orally given standardized *E. longifolia* extract at a dose of 50 mg/kg for 28 days, consecutively. Blood samples (∼0.15 mL at pre-treatment and day 1; 0.2 mL at day 7, 14, 21, and 28) were collected from the tail veins of treated rats in accordance to the guidelines set by the John Hopkins Medicine Guidelines for multiple blood draws (John Hopkins Medicine, 2012). Sera were separated through centrifugation at 3,000 rpm for 15 min and stored at -20°C prior to analysis.

### Protein Estimation of Serum Samples

The protein concentrations of each samples group (*n* = 6) were quantified using 660 nm Protein Assay kit (Thermoscientific, Rockford, IL, USA). The serum samples were diluted in 1:1000 factor and then added into microplate wells (10 μl each). One hundred fifty micro liter of the protein assay reagent was added into each well and then incubated for 5 min. The absorbance was measured using μQuant^TM^ microplate spectrometer.

### Protein Separation Using 2-DE

Six biologically replicates for each treatment (pre-treatment, days 1, 7, 14, 21, and 28) were analyzed by 2-DE (Chen et al., 2008; Chen et al., 2009; Mu et al., 2012). Briefly, the serum samples (∼3 μl each) were rehydrated in 11 cm precast Immobilline Drystrips at pH 4–7 (GE Healthcare Biosciences, Uppsala, Sweden) for 16 h. The rehydrated strips were subjected to isoelectric focusing with IPGphor 3 (GE Healthcare Biosciences, Uppsala, Sweden) for a total duration of 14 kV/h (Phase 1: 500 V, 50 mA, 500 V/h; Phase 2: 1,000 V, 50 mA, 1,000 V/h; Phase 3: 8,000 V, 50 mA, 12.5 V/h). Focused strips were equilibrated with equilibration buffer (1.5 M Tris-HCl, pH8.8; 6 M urea; 2% w/v SDS; 30% v/v glycerol) containing 0.06 M DTT for 15 min. They were further incubated in equilibration buffer containing 4.5% v/v iodoacetamide for 15 min. For the second dimension, the focused strips were subjected to sodium dodecyl sulfate–polyacrylamide gel electrophoresis (SDS–PAGE) using 8–18% gradient gels. According to the optimized protocol (Phase 1: 50 V, 17 mA, 15 W for 30 min; Phase 2: 600 V, 25 mA, 15 W for 2 h), electrophoresis was performed using the SE 600 Ruby Electrophoresis System and Power supply EPS601 (GE Healthcare, Uppsala, Sweden).

### Staining of 2-DE Gels

After 2-DE protein separation, the gels were fixed and silver-stained according to Mortz et al. (2001). For protein identification by tryptic digestion and MS, a modified silver-staining method was performed according to Shevchenko et al. (1996).

### Analysis of Protein Profiles

The silver-stained gels were scanned using the ImageScanner III (GE Healthcare Biosciences, Uppsala, Sweden). The Image Master Platinum 7.0 software (GE Healthcare Biosciences, Uppsala, Sweden) was used to evaluate the 2-DE profiles of serum samples obtained from all the six rats pre-treated and treated with *E. longifolia*. The percentage volume contribution of protein spots was calculated (i.e., the spot volume of a protein expressed as a percentage of the total spot volume of all detected proteins).

### Statistical Analysis

All values were expressed as mean ± SD. The Student’s *t*-test was used to compare the experimental groups with regard to mean percentage volume contribution for the detected spots. Student’s *t*-test was also used to compare the mean percentage inhibition of AHS following the treatment at various intervals compared to that of pre-treatment. A *p*-value of <0.05 was considered significant.

### Protein Identification Using MALDI TOF/TOF

For MALDI-TOF-MS, the gels plugs containing proteins of interest from the 2-DE gels were subjected to in-gel digestion based on the manufacturer’s instruction (Pierce, Rockford, IL, USA). The MS analysis was performed using Applied Biosystem 4800 Proteomic analyzer. Mass spectra data were submitted to the Mascot search engine (Matrix Science Ltd, London, UK) for protein identification against *Rattus* entries in the NCBI database (accessed on 15 Nov 2012). Proteins were considered positively identified as described by Delahunty and Yates (2005).

### Competitive Enzyme-linked Immunosorbent Assay (ELISA)

Decreased expression of alpha-2-HS glycoprotein (AHS) was validated using a modified version of the competitive ELISA as previously described (Mu et al., 2013). Serum samples (*n* = 6 for each group; pre-treatment, days 1, 7, 14, 21, and 28) were diluted with 0.05 M sodium bicarbonate buffer (pH 9.6) and loaded into microtiter plates (Nunc, Denmark) for overnight coating of wells at 4°C. Plates were washed three times with 100 μl of PBST (phosphate-buffered saline [PBS] with 0.05% [v/v] Tween-20), and the wells were subsequently blocked with 100 μl of 0.5% gelatin in PBST for 30 min at room temperature. After blocking, the plates were washed with PBST and then incubated with goat anti-rat AHS (1:1,000 in PBST) for 1 h at room temperature (Santa Cruz Biotechnology, Santa Cruz, CA, USA). Positive controls were prepared without serum, and blanks contained washing buffer. After incubation, the wells were washed with PBST, and 200 μl of horseradish peroxidase (HRP)-labeled secondary anti-goat IgG (1:5,000) was added to each well. The plate was then incubated for 1 h at room temperature, followed by washing with PBST. 100 μl of 3,3′,5,5′-tetramethylbenzidine substrate (Sigma–Aldrich Company, St. Louis, MO, USA) was added to each well and the plate was incubated for 15 min at room temperature in the dark, followed by reaction termination (100 μl 2 M sulphuric acid per well). Absorbance was read at 450 nm using an ELISA plate reader. The amount of AHS was determined by the percentage inhibition of substrate hydrolysis as described previously (Reddington et al., 1991).

## Results

### Determination of Serum Protein Concentration

The concentrations of serum protein samples were determined using a protein estimation kit. Estimation of the protein concentration was based on the preparation of several dilutions of BSA as the standard. All samples have protein concentration with variation not more than 25% (**Table 1**), which indicated that their protein amounts were almost equal.

**Table 1:** Concentration of protein in serum samples.

Serum samples (Day)	Concentration of serum protein (μg/μl)
Pre-treatment	52.918 ± 14.554
1	61.665 ± 40.647
7	66.250 ± 31.134
14	50.415 ± 22.827
21	75.418 ± 28.199
28	64.168 ± 30.138

*All values were expressed as mean ± SD; n = 6*.

### 2-DE Profile of Rat Serum Samples

**FIGURE 2:**
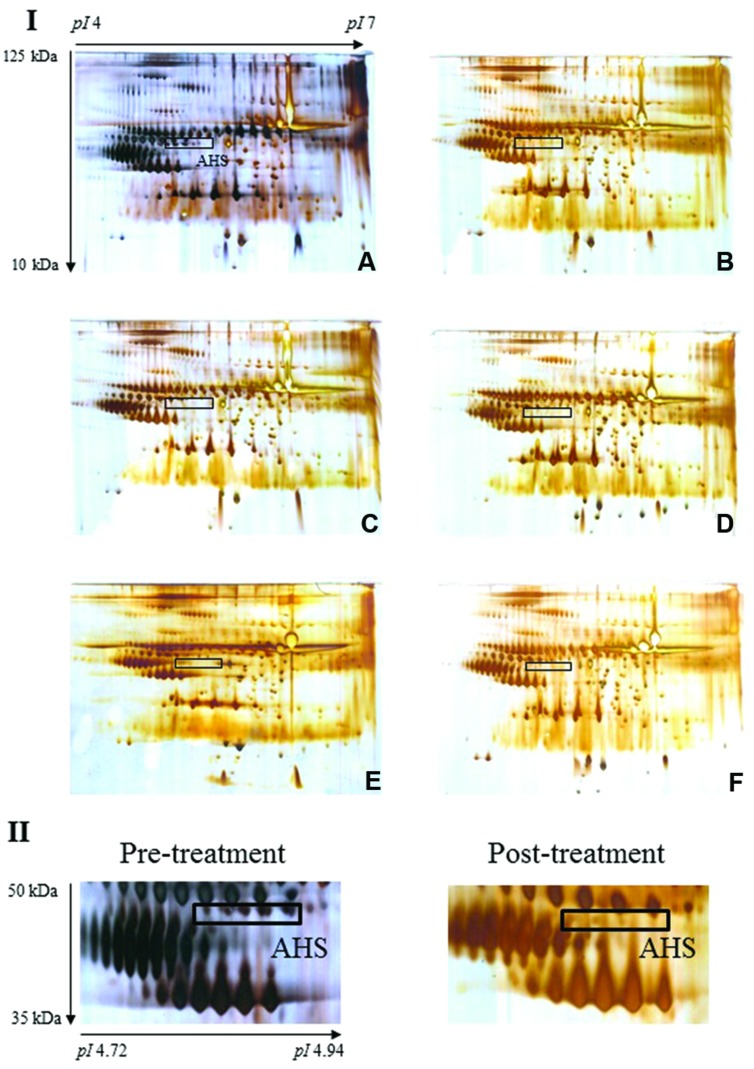
**Typical 2-DE profiles of rat serum samples**. (A–F) of section I illustrate representative 2-DE profiles of rat serum from pre-treatment and days 1, 7, 14, 21, and 28, respectively. The boxed protein cluster corresponds to AHS, which was lost in the serum samples from rats treated with *E. longifolia* extract (section II displays cropped images). Gel acidity and relative molecular weights are indicated.

Serum proteins from the pre-treatment and treated (days 1, 7, 14, 21, and 28) rat groups were separated by 2-DE on the basis of their isoelectric points (pIs) and molecular weights, yielding complex protein profiles consisting of hundreds of highly-resolved protein spots. **Figure 2** illustrates representative serum protein profiles of rats before and after treatment with *E. longifolia* extract.

### Image Analysis of Rat Serum Proteins

Image analysis was performed on the silver-stained 2-DE gels obtained from each serum sample of pre-treatment and extract-treated rats. Notably, expression of most of the detected proteins was comparable with the exception of a protein cluster designated AHS. Protein spots corresponding to AHS were present in pre-treatment sera but were barely visible in treated samples. **Table 2** displays the average percentage of volume contribution of AHS detected in pre-treatment sera.

**Table 2:** Mean percentage of volume contribution of AHS in pre-treatment sera.

Serum protein	Mean % Vol ±*SD*	*p*^∗^
AHS	0.705 ± 0.183	0.00025

*^∗^denotes significant value relative to the expression of days 1, 7, 14, 21, and 28, respectively*.

### Identification of the Differentially Expressed Rat Serum Protein

Analysis of the differential protein cluster with MALDI-TOF MS/MS revealed more than twenty peaks with different *m/z*, and submission of the MS data to the Mascot search engine allowed confident identification of AHS. **Table 3** shows the data for mass spectrometric identification of AHS after searching the NCBI database.

**Table 3:** Identification of the differentially expressed rat serum protein detected on 2-DE gel.

Protein entry name^+^	Protein name	Accession number^#^	Nominal mass (kDa)/pI	MOWSE protein score	Sequence coverage (%)
AHS	Alpha-2-HS glycoprotein	gi|6978477	38/6.30	64	9

*(a) + Protein entry name is from the UniProtKB database. (b) # Accession number is from the NCBI database using Mascot search engine (accessed on 15 Nov 2012)*.

### Competitive ELISA

**FIGURE 3:**
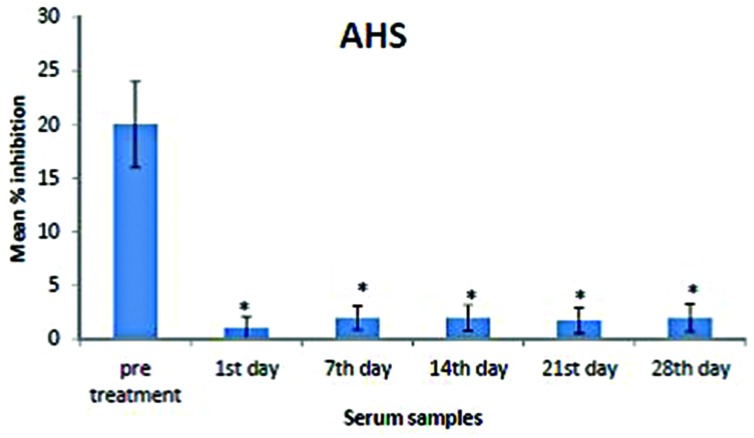
**Mean percentage of inhibition of AHS in the pre-treatment and post-treatment serum samples**. Analysis was performed by competitive ELISA. AHS concentrations in the pre-treatment sera were significantly higher than those in the post-treatment samples (days 1, 7, 14, 21, and 28). The amount of AHS present was proportional to the percentage inhibition of substrate hydrolysis. Asterisk denotes statistical significance (*p <* 0.05) of post-treatment samples compared to that of pre-treatment sample (*n* = 6).

To validate AHS reduction upon extract treatment, competitive ELISA was performed on the serum samples (*n* = 6 for each group; pre-treatment, days 1, 7, 14, 21, and 28) using polyclonal antisera against AHS. We observed that AHS expression was significantly higher in pre-treatment sera compared to post-treatment sera (**Figure 3**).

## Discussion

Here, we have analyzed serum from rats treated with standardized *E. longifolia* extract by 2-DE and observed the obvious loss of a particular protein cluster upon treatment. Subsequently, we identified this protein cluster to be AHS, which could be seen in sera from untreated rats but was hardly detectable in sera from rats treated with standardized *E. longifolia* extract for 1–28 days. Finally, we confirmed the extract-induced reduction in AHS using competitive ELISA.

Alpha-2-HS glycoprotein, also known as alpha2-Heremans-Schmid glycoprotein or fetuin A, is a serum protein that participates in bone metabolism since it is one of the major components of non-collagenous bone matrix (Dickson et al., 1975). It accumulates in the skeleton due to its high affinity for hydroxyapatite. Also, several reports have suggested that AHS is involved in host defense, particularly in phagocytosis and innate immunity (Arnaud et al., 1988). Indeed, AHS is a negative acute phase protein and its concentration decreases during infection.

In addition, AHS has been linked to insulin resistance and diabetes (Stefan et al., 2006). The insulin receptor is a disulfide-linked heterotetrameric protein complex consisting of two hormone-binding α subunits and two signaling β subunits containing tyrosine kinase activity (Srinivas et al., 1993). Insulin binds to this receptor located on the surface of insulin responsive cells, leading to the activation of the tyrosine kinase and subsequently the autophosphorylation of β subunit in order to activate the function of insulin toward the physiological function (Myers and White, 1996). The phosphorylated form of AHS acts as a natural competitve inhibitor of insulin, which blocks both activities, potentially contributing to the development of type-2 diabetes (Srinivas et al., 1993). It was also reported that testosterone levels correlated positively with insulin sensitivity (Pitteloud et al., 2005). Thus, testosterone, which can be produced in response to insulin, is a modulator of insulin sensitivity. Therefore, we propose that the quassinoid-containing *E. longifolia* extract affects male infertility by suppressing AHS expression, which indirectly increases insulin sensitivity and testosterone levels in accordance with our previous findings (Low et al., 2013). Our earlier study showed that the testosterone enhancement activity of *E. longifolia* has been prolonged in the treated rats after the 28 days treatment of *E. longifolia* extract when they had displayed significantly higher sperm count (*P* < 0.001) than that of the untreated control (Low et al., 2013). In addition, the present finding also proposed that the *E. longifolia* extract could be used as a supplement to prevent insulin resistance by modulating phosphorylated AHS, which is present in healthy individuals (Haglund et al., 2001).

Taken together, our findings indicate that serum AHS was reduced in rats treated with standardized *E. longifolia* extract, and provide rational for further investigation into the effects of this natural extract in animal models involving infertility and diabetes.

## Author Contributions

YC, B-SL, and K-LC contributed to the design of the study and drafting the manuscript. W-MP and SS participated in the data analysis and critically revised the manuscript. AK-W and C-KC carried out the experiments and analyzed the data. All authors read and approved the final manuscript.

## Conflict of Interest Statement

The authors declare that the research was conducted in the absence of any commercial or financial relationships that could be construed as a potential conflict of interest.
